# Hyper Accumulation of Arsenic in Mutants of *Ochrobactrum tritici* Silenced for Arsenite Efflux Pumps

**DOI:** 10.1371/journal.pone.0131317

**Published:** 2015-07-01

**Authors:** Tânia Sousa, Rita Branco, Ana Paula Piedade, Paula V. Morais

**Affiliations:** 1 IMAR-CMA, Coimbra, Portugal; 2 CEMUC-Department of Mechanical Engineering, University of Coimbra, 3030–788 Coimbra, Portugal; 3 Department of Life Sciences, University of Coimbra, Coimbra, Portugal; University of Ottawa, CANADA

## Abstract

*Ochrobactrum tritici* SCII24^T^ is a highly As-resistant bacterium, with two previously described arsenic resistance operons, *ars1* and *ars2*. Among a large number of genes, these operons contain the *arsB* and *Acr3* genes that encode the arsenite efflux pumps responsible for arsenic resistance. Exploring the genome of *O*. *tritici* SCII24^T^, an additional putative operon (*ars3*) was identified and revealed the presence of the *Acr3_2* gene that encodes for an arsenite efflux protein but which came to prove to not be required for full As resistance. The genes encoding for arsenite efflux pumps, identified in this strain, were inactivated to develop microbial accumulators of arsenic as new tools for bioremediation. Six different mutants were produced, studied and three were more useful as biotools. *O*. *tritici* wild type and the *Acr3*-mutants showed the highest resistance to As(III), being able to grow up to 50 mM of arsenite. On the other hand, *arsB*-mutants were not able to grow at concentrations higher than 1 mM As(III), and were the most As(III) sensitive mutants. In the presence of 1 mM As(III), the strain with *arsB* and *Acr3_1* mutated showed the highest intracellular arsenic concentration (up to 17 ng(As)/mg protein), while in assays with 5 mM As(III), the single *arsB*-mutant was able to accumulate the highest concentration of arsenic (up to 10 ng(As)/mg protein). Therefore, *arsB* is the main gene responsible for arsenite resistance in *O*. *tritici*. However, both genes *arsB* and *Acr3_1* play a crucial role in the resistance mechanism, depending on the arsenite concentration in the medium. In conclusion, at moderate arsenite concentrations, the double *arsB*- and *Acr3_1*-mutant exhibited a great ability to accumulate arsenite and can be seen as a promising bioremediation tool for environmental arsenic detoxification.

## Introduction

Arsenic is a natural metalloid widely distributed in air, water and soil, and is considered as one of the chemicals of major public health concern [1]. Arsenic occurs in the environment in four oxidation states, but the most common forms are the soluble species arsenate [AsO_4_^3-^] and arsenite [AsO_2_^-^]. Arsenate is a structural analogue of phosphate that can enter cells via phosphate membrane transport systems, disrupting the metabolic processes that require oxidative phosphorylation [2,3]. Arsenite, the most toxic of arsenic oxyanions, is transported into cells by aqua-glyceroporins and exerts its toxicity by binding to thiol groups (SH) in proteins, impairing their function [2,3]. Many organisms like bacteria have developed a variety of mechanisms that allow them to grow in environments contaminated with arsenic. Such mechanisms include (i) arsenite methylation; (ii) arsenite oxidation to arsenate; (iii) arsenite extrusion systems; or (iv) arsenate reduction and consequent extrusion of arsenite [3].

One of the best documented mechanisms of arsenic resistance is the arsenite efflux from cells, performed by two different and unrelated families of membrane transport proteins, ArsB and Acr3p [4,5]. ArsB is encoded by the *arsB* gene that in some cases, is rearranged in operons with three genes (*arsRBC*), where ArsB alone extrudes arsenite with energy supplied by the membrane potential of the cell [6,7]. In other cases, *arsB* is clustered in operons with five genes (*arsRDABC*), where ATPase ArsA hooks up with ArsB and then arsenite is pumped out in an ATP-dependent process [6,8]. The Acr3p family includes the *arsB* gene of *Bacillus subtilis* [9] and the *Acr3* gene from *Saccharomyces cerevisiae* [10], and encodes a membrane protein that catalyzes the extrusion of arsenite from cytosol [4]. Although ArsB and Acr3p share the same function, these proteins do not show sequence similarities.

*Ochrobactrum tritici* SCII24^T^ has been reported as a strain with high capacity to resist to arsenic toxicity, able to grow in the presence of As(III) up to 50 mM [11]. Previous works identified two chromosomally located arsenic resistance operons: operon *ars1* that contains five genes encoding proteins ArsR, ArsD, ArsA, cystathionine β-synthase (CBS)-domain-containing protein and ArsB, and confers resistance to arsenite and antimonite; and operon *ars2*, responsible for the resistance to arsenite and arsenate, composed of six genes encoding two additional ArsR, two ArsC, one ACR3 and an ArsH-like protein [11]. Additionally, this strain is capable of arsenite oxidation through the arsenite oxidase designated AioAB [12]. In this bacterium, the presence of different mechanisms for coping with arsenic in addition with other intrinsic characteristics such as lack of pathogenicity, high growth rates, no-exigent growth requirements and stability of the genome, makes it an interesting organism to be manipulated for the development of potential biotools [11,12].

The use of biotools to remove arsenic from polluted environments has been gaining notice to interest because of their potential in providing an effective technology for arsenic remediation. Moreover, biological approaches are often more environmentally friendly and economically viable [13]. Several approaches have been developed to deal with arsenic-contaminated waters including the use of bioaccumulation or biosequestration abilities of organisms, and the development of biosorbents [14,15].

In this work, the genome of strain *O*. *tritici* SCII24^T^ was explored in order to reveal additional arsenic resistance related genes allowing a better understanding of the mechanisms of this organism to cope with arsenic. The main objective of this work was to construct an *O*. *tritici* mutant with potential for bioremediation applications, with special abilities to resist and accumulate arsenic. The genes that encode for arsenite efflux pumps were inactivated to achieve strains able to accumulate arsenic. With this strategy, we aim to obtain an efficient bioremediation tool able to remove arsenite from the environment.

## Materials and Methods

### Bacterial strains, plasmids and growth conditions

Bacterial strains and plasmids used in this study are listed in Table 1. *O*. *tritici* SCII24^T^ strains and *Escherichia coli* AW3110 (7) were grown aerobically at 35°C in Luria-Bertani (LB) medium containing 10 g/L tryptone, 5 g/L yeast extract and 5 g/L NaCl or in Chemically Defined Medium (CDM) composed by 6.06 g/L Tris, 4.68 g/L NaCl, 1.49 g/L KCl, 1.07 g/L NH_4_Cl, 0.43 g/L Na_2_SO_4_, 0.2 g/L MgCl_2_.6H_2_O, 0.03 g/L CaCl_2_.2H_2_O, 0.23 g/L Na_2_HPO_4_.12H_2_O and 0.3% glucose. These media were supplemented with corresponding antibiotics, ampicillin (100 μg/mL), gentamicin (15 μg/mL), hygromycin (200 μg/mL) and sucrose (5%) when required. *E*. *coli* S17-1 and *E*. *coli* DH5α strains were used as host for the cloning vectors.

**Table 1:** Bacterial strains and plasmids used in this work.

Strain or Plasmid	Relevant Characteristic(s)	Reference or Source
**Bacterial Strains**		
* O*. *tritici* SCII24^T^	Type strain; Amp^r^; As(III)^r^; As(V)^r^; Sb(III)^r^	LMG
* arsB* mutant	Single mutant of SCII24^T^; Gm^r^; *arsB* mutated	This study
* Acr3_1* mutant	Single mutant of SCII24^T^; Hyg^r^; *Acr3_1* mutated	This study
* Acr3_2* mutant	Single mutant of SCII24^T^; *Acr3_2* mutated	This study
* arsB/Acr3_2* mutant	Double mutant of SCII24^T^; Gm^r^; *arsB* and *Acr3_2* mutated	This study
* arsB/Acr3_1* mutant	Double mutant of SCII24^T^; Gm^r^; Hyg^r^; *arsB* and *Acr3_1* mutated	This study
* arsB/Acr3_1/Acr3_2* mutant	Triple mutant of SCII24^T^; Gm^r^; Hyg^r^, *arsB*, *Acr3_1* and *Acr3_2* mutated	This study
* E*. *coli* S17-1	Conjugation donor strain	[34]
* E*. *coli* DH5-α	λ^−^ ϕ80d*lacZ*°M15 °(*lacZYA-argF*)*U169 recA1 endA1 hsdR17*(r_k_^−^ m_k_^−^) s*upE44 thi-1 gyrA relA1*	Promega
* E*. *coli* AW3110	K12 F^-^ IN(*rrnD-rrnE*) Δ*ars*::cam	[7]
**Plasmids**		
pK18::mob	pUC18 derivative; lacZα Kan^r^; mob site; suicide vector	[35]
pTE-10M0X	pTE-MCS derivative; Hyg^r^	Addgene
pBBR1MCS-5	pBBR1MCS derivative; Gm^r^	[17]
pGEM-T Easy	Amp^r^; T-tailed PCR product cloning vector; lacZ	Promega
pJQ200SK	Suicide vector; *sacB*; Gm^r^	ATCC
p*Acr3*	pJQ200SK derivative carrying the gene *Acr3_1*	This study
p*Acr3* **::**Hyg	pJQ200SK derivative carrying the gene *Acr3_1* disrupted by the insertion of Hyg^r^ cassette	This study
p*arsB*	pK18::mob derivative carrying the gene *arsB*	This study
p*arsB*::Gm	pK18::mob derivative carrying the gene *arsB* disrupted by the insertion of Gm^r^ cassette	This study
p*Acr3_2*	pJQ200SK derivative carrying the upstream and the downstream fragments of the gene *Acr3_2*	This study

LMG, Laboratorium voor Microbiologie, Universiteit Gent.

ATCC, American Type Culture Collection.

### Sequencing of O*chrobactrum tritici* SCII24^T^ genome

*O*. *tritici* SCII24^T^ was isolated and the purified DNA was directly sequenced using Illumina (California, USA). Raw data was assembled using the assembly program Edena V3, and assembly coverage >8 was the standard for the assessment of high quality assembly. The assembled contigs were submitted to the RAST (Rapid Annotation using Subsystems Technology) annotation server for subsystem classification and functional annotation [16]. By using RAST, genes were annotated according to functional domains, recognized in multiple databases, grouped in functional subsystems, and divided in hypothetical and non-hypothetical genes. Arsenic related coding sequences (CDSs) were assigned using BLASTp with KEGG Orthology (KO). The nucleotide sequence of operon *ars3*, sequenced in this work, has been deposited in the GenBank database under accession number KP214556.

### Construction of *Ochrobactrum tritici* SCII24^T^ mutants

Bacterial strains and plasmids used in this work are indicated in Table 1. The first single *arsB* mutant was constructed by disruption of the *arsB* gene through insertion of a gentamycin resistance cassette (Gm^r^) into its unique *Not*I restriction site. Therefore, *arsB* gene was amplified by PCR from the type strain *O*. *tritici* SCII24^T^ using the specific primers containing additional *Xba*I/*Hin*dIII recognition sites, xbaarsBf—TCTAGAATGCTGGCTGCCCTGCTGAT and hindarsBr—AAGCTTTCAGACAACACTGAGTCTCA. The PCR product (≈ 1300 bp) was digested with *Xba*I and *Hin*dIII enzymes, and cloned into the suicide vector pK18mob previously digested with the same enzymes, resulting in the plasmid p*arsB*. The Gm^r^ cassette (≈ 900 bp) was amplified from plasmid PBBR1MCS-5 [17] using the primers that incorporated the *Not*I recognition site. The obtained fragment was then digested with *Not*I enzyme and ligated to p*arsB*, generating the vector p*arsB*::Gm. This plasmid was transformed into *E*. *coli* S17-1 and transferred to the recipient strain *O*. *tritici* SCII24^T^ by biparental conjugation using the filter mating method [18]. Double-crossover transconjugants were selected on LB plates with ampicillin and gentamicin. Positive mutants were confirmed by PCR using the specific primers used to amplify the *arsB* and the Gm^r^ genes. Single *Acr3_1* mutant was constructed by disruption of the *Acr3_1* gene through insertion of a hygromycin resistance cassette (Hyg^r^) into its unique *Pst*I restriction site. The *Acr3_1* gene was amplified by PCR from the type strain *O*. *tritici* SCII24^T^ using specific primers, xbaAcr3f—TCTAGAAGTTCCACTTTCGAAC and hindAcr3r—AAGCTTTCAGAGTTTTCCTGTTTCGC. The PCR product (≈ 1000 bp) was cloned into pGEM-T Easy vector and digested with *Not*I. The released *Acr3_1* fragment was then cloned into *Not*I site of the suicide vector pJQ200SK, resulting in the plasmid p*Acr3_1*. The Hyg^r^ cassette (≈ 1500 bp) was amplified from plasmid pTE-10M0X with primers that incorporated the *Pst*I recognition site. The obtained fragment was digested with *Pst*I enzyme and ligated to p*Acr3_1*, generating the plasmid p*Acr3_1*::Hyg. This construct was subsequently transformed into *E*. *coli* S17-1 and transferred to the recipient strain *O*. *tritici* SCII24^T^ by biparental conjugation. Double-crossover transconjugants were selected on LB plates with ampicillin, hygromycin and sucrose. Positive mutants were confirmed by PCR using the specific primers to amplify the *Acr3_1* and Hyg^r^ genes. Single *Acr3_2* mutant was obtained by removing part of the newly annotated gene *Acr3_2*. In order to do it, the initial gene portion of 300bp amplified by specific primers, xbaAcr3’up-f—TCCTCTAGACGGCGTGCTTCTCGGCACAGT and pstAcr3’up-r—GACCTGCAGAGGAAGAACCAAGCGTAAACG and the terminal gene portion of 300 bp amplified using the specific primers, pstAcr3’down-f—GACCTGCAGCGATCACGCTGGCTGCGCTGC and xhoAcr3’down-r—GACCTCGAGACTGGAACCTCAACGAGAGGA were digested with the pair of enzymes respective. These fragments were cloned into pJQ200sk vector at the *Xba*I/*Pst*I and *Pst*I/*Xho*I restriction sites, originating the construct p*Acr3_2*. This plasmid was subsequently transformed into *E*. *coli* S17-1 and transferred to the recipient strain *O*. *tritici* SCII24^T^, and the transconjugants were selected on LB plates with ampicillin and sucrose. Positive mutants were confirmed by PCR using the specific primers to amplify the *Acr3_2* gene. Double *arsB/Acr3_2* mutant was constructed in order to disrupt both *arsB* and *Acr3*_2 genes. The p*Acr3_2* plasmid was transformed into *E*. *coli* S17-1 and transferred to the recipient *Acr3_2* mutant. Transconjugants were selected on LB plates with ampicillin, gentamicin and sucrose. Positive mutants were confirmed by PCR using the specific primers to amplify the *arsB* and *Acr3_2* genes. Double *arsB/Acr3_1* mutant was constructed using the previous strategies to disrupt both *arsB* and *Acr3_1* genes. Thus, suicide plasmid p*arsB*::Gm transformed in *E*. *coli* S17-1 was used to conjugate with the recipient strain *Acr3_1* mutant. Transconjugants were selected on LB plates with ampicillin, hygromycin and gentamicin. Positive mutants were confirmed by PCR. Triple *arsB/Acr3_1/Acr3_2* mutant was created by disrupting all three efflux bombs, *arsB*, *Acr3_1* and *Acr3*_2. The p*Acr3_2* plasmid was transformed into *E*. *coli* S17-1 and transferred to the recipient *arsB/Acr3_1* mutant. Transconjugants were selected on LB plates with ampicillin, hygromycin, gentamicin and sucrose, and positive mutants were confirmed by PCR with specific primers to amplify the three genes.

### Arsenite resistance assays

Arsenite resistance was determined by the clonogenic assay. Mutated and non-mutated *O*. *tritici* strains were grown in LB to an O.D. 600 nm of 0.2. Serial dilutions of each culture were prepared in NaCl 0.85% and were plated in triplicate on LB plates supplemented with increasing concentrations of As(III) [from 0 mM to 50 mM As(III)] and with the respective antibiotics. Plates were incubated at 35°C, and colonies were counted after 2–3 days. Differences between mutants and wild type *O*. *tritici* were also verified in Petri dishes, by evenly swabbing the cultures on agar plates with corresponding antibiotics, and pressing filter discs saturated with increasing arsenite concentrations (0 mM, 100 mM, 250 mM and 500 mM). After 2 days of incubation, differences in the arsenite sensitivity of the strains were evaluated by analysis of the growth inhibition halos around the discs.

### Arsenic uptake assays

The uptake assays were made by exposing the strains to arsenic in the form of As(III) or As(V). In the arsenate uptake assays, *E*. *coli* AW3110, a strain non-resistant to arsenic (7), was used as control. Briefly, overnight-grown cultures of wild *O*. *tritici* SCII24^T^ or As-mutants and *E*. *coli* AW3110 were diluted in 250 mL of a new LB medium supplemented with the respective antibiotics, and grown to exponential phase (± 0.5 O.D. 600 nm). Cultures were harvested by centrifugation at 4000 rpm for 30 min, and resuspended in 250 mL of CDM. Uptake assays were initiated by adding 1 mM As(III), 5 mM As(III) or 5 mM As(V) to the cellular suspensions, followed by incubations at 35°C, 150 rpm for 3 hours. After incubation, bacterial suspensions were harvested by centrifugation, washed twice with ice cold PBS (8 g/L NaCl, 0.2 g/L KCl, 1.44 g/L Na_2_HPO_4_, 0.24 g/L KH_2_PO_4_, pH 7.4) and resuspended in 2 mL of H_2_O:HNO_3_ 10% (1:1). Cells were disrupted by freezing at -20°C for 10 min, followed by heating at 50°C for 60 to 120 min until no clump was observed, and then cells were centrifuged at 4000 rpm for 30 min, at 4°C. The supernatants were collected into new centrifuge tubes and used for the arsenic measurements. Resultant pellets were dissolved in 1 mL of NaOH 0.5 M, heated at 37°C for 30 min, and used for protein quantification. Total arsenic was analyzed by Inductively Coupled Plasma Mass Spectrometry (ICP-MS) in an ICP-MS Thermo X Series, and total protein was determined using the Bradford assay [19]. Intracellular arsenic content was expressed as nanogram of As per microgram of total cellular protein. Accumulation of arsenite inside the cells was also evaluated by Scanning Electron Microscopy (SEM) and Electron Probe Microanalysis Analysis (EPMA). For this procedure, exponentially grown cells were incubated for 3 hours in a CDM medium contaminated with 1 mM As(III) or 5 mM As(V), as described above. Then, cells were collected and placed onto an appropriate stainless steel support. Cells were dried, fixed with glutaraldehyde 2.5%, and dehydrated with increasing concentrations of ethanol solutions (70, 80, 90, 95 and 100%). After complete evaporation of the solvent, samples were sputtered coated with 30 nm of gold for SEM observations, secondary electron mode, in a Philips XL30 equipment with 10 kV beam. The same samples were used to perform elemental map distribution of As, in a 50x50 μm^2^ area, in a Cameca, Camebax SX50 EPMA equipment with 10 kV beam.

## Results

### *Ochrobactrum tritici* SCII24^T^ genome sequencing and genes annotation

**Fig 1:**
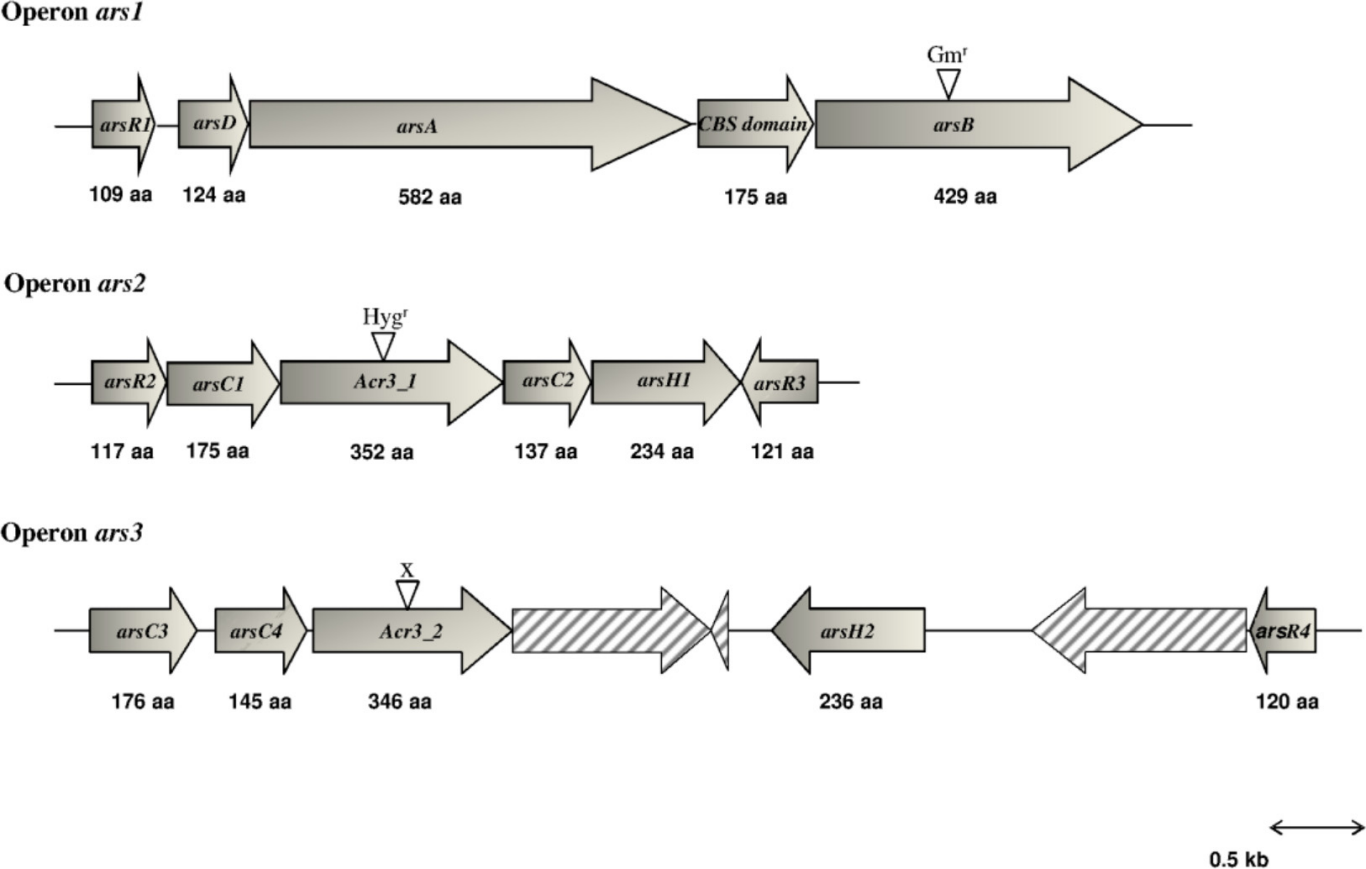
Genetic organizations of the three *ars* operons present in strain *Ochrobactrum tritici* SCII24^T^. Gene orientations are shown by arrows. Pattern field arrows are representative of putative genes with unknown functionality. Mutation sites are indicated by inverted triangles. Gm^r^ and Hyg^r^ mean the insertion of gentamycin and hygromycin resistance cassettes, respectively and X means deletion of partial gene sequence.

The genome of *O*. *tritici* SCII24^T^ was sequenced using Illumina. Annotation of the genes confirmed the presence of the arsenic resistance genes *arsB* and *Acr3_1* already identified in this strain, included in two different chromosomally located arsenic resistance operons [11]. Besides these two arsenic efflux systems, it was also possible to identify a third possible operon probably involved in arsenic resistance that was named putative operon *ars3* (Fig 1). This one, detected in a contig carrying *ParA*, *IncI*, *IncF* and *repC* genes, comprised nine open reading frames (ORFs) and five of them showed high homology with genes already found in arsenic resistance determinants and is probably plasmid located. The first gene of this genetic region, *arsC3*, encoded a predicted protein of 176 aa with 98% identity and 96% similarity to an ArsR from *Agrobacterium tumefaciens* (WP_020810063), and also with 84% identity and 94% similarity to an ArsC from *A*. *tumefaciens* (CDN92172). The second gene, *asrC4*, encoded a protein of 145 aa with 96% identity and 96% similarity to an ArsC from *A*. *tumefaciens* (WP_020810062). The next gene, *Acr3_2*, encoded a protein of 346 aa with 95% identity and 96% similarity to an arsenic resistance protein ArsB from *A*. *tumefaciens* (WP_020810061). Downstream, the gene *arsH2* was found, encoding a protein of 236 aa with 94% identity and 97% similarity to a NADPH-dependent FMN reductase from *A*. *tumefaciens* (WP_020810058). The last gene of the putative operon *ars3* was *arsR4*, which encoded a protein of 120 aa with 91% identity and 94% similarity to an ArsR from *A*. *tumefaciens* (WP_020810055). Alignment of these new genes with those from operons *ars1* and *ars2* revealed that the currently identified *arsR4* showed higher homology with *arsR3* (70% similarity) than with the other previously identified regulators of the strain. Also, *arsC3* was more similar to *arsC1* (76% similarity) while *arsC4* was more similar to *arsC2*. On the other hand, *arsH2* was very similar to *arsH1*, exhibiting 72% of homology. Therefore, the putative operon *ars3* showed an arrangement very similar to the operon *ars2*.

### Arsenite resistance assays

**Fig 2:**
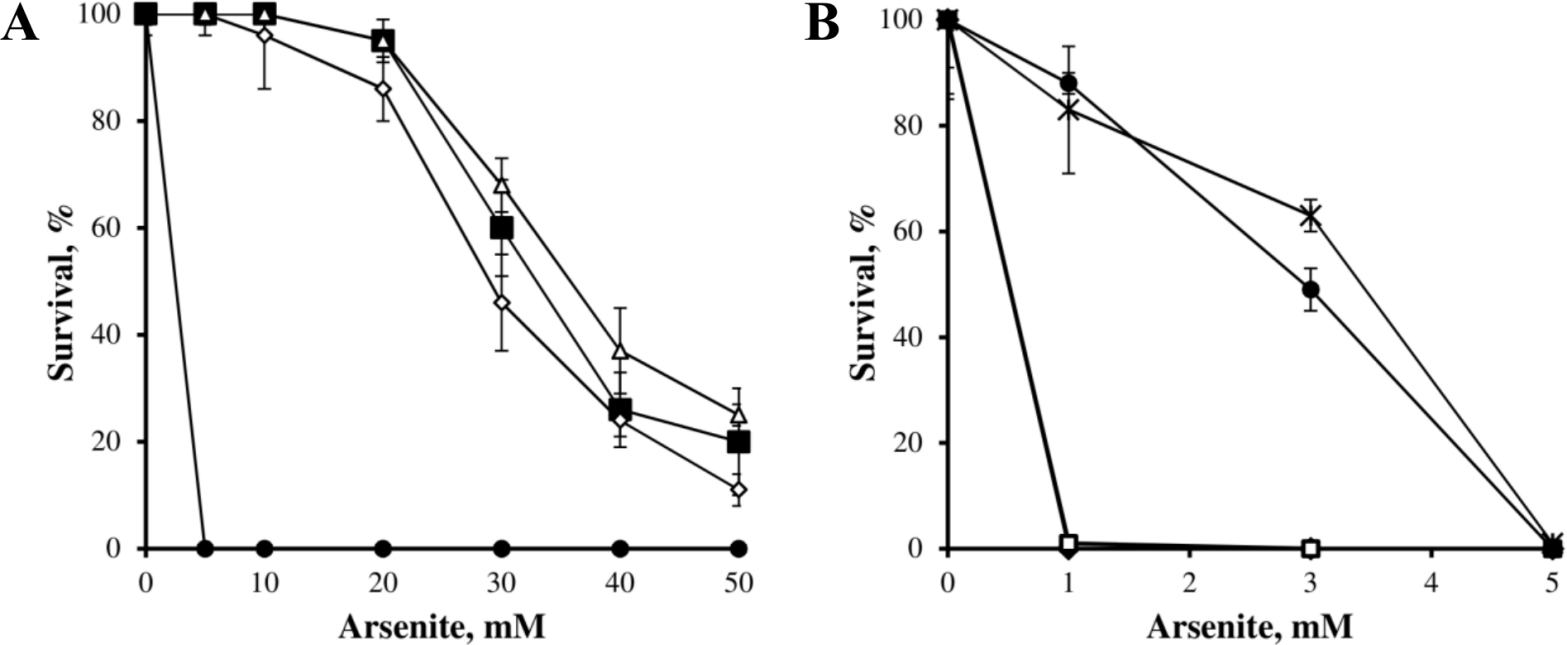
Clonogenic survival at different arsenite concentrations of strains *O*. *tritici* SCII24 (■), *arsB* mutant (●), *Acr3_1* mutant (Δ), *Acr3_2* mutant (◊), *arsB/Acr3_2* mutant (*), *arsB/Acr3_1* mutant (◆) and *arsB/Acr3_1/Acr_2* mutant (□). The cultures were spread on LB plates contaminated with different As(III) concentrations and the colonies were counted after 2–3 days of incubation at 35°C. Data shown are the mean values (± standard deviations) obtained from three independent experiments.

**Fig 3:**
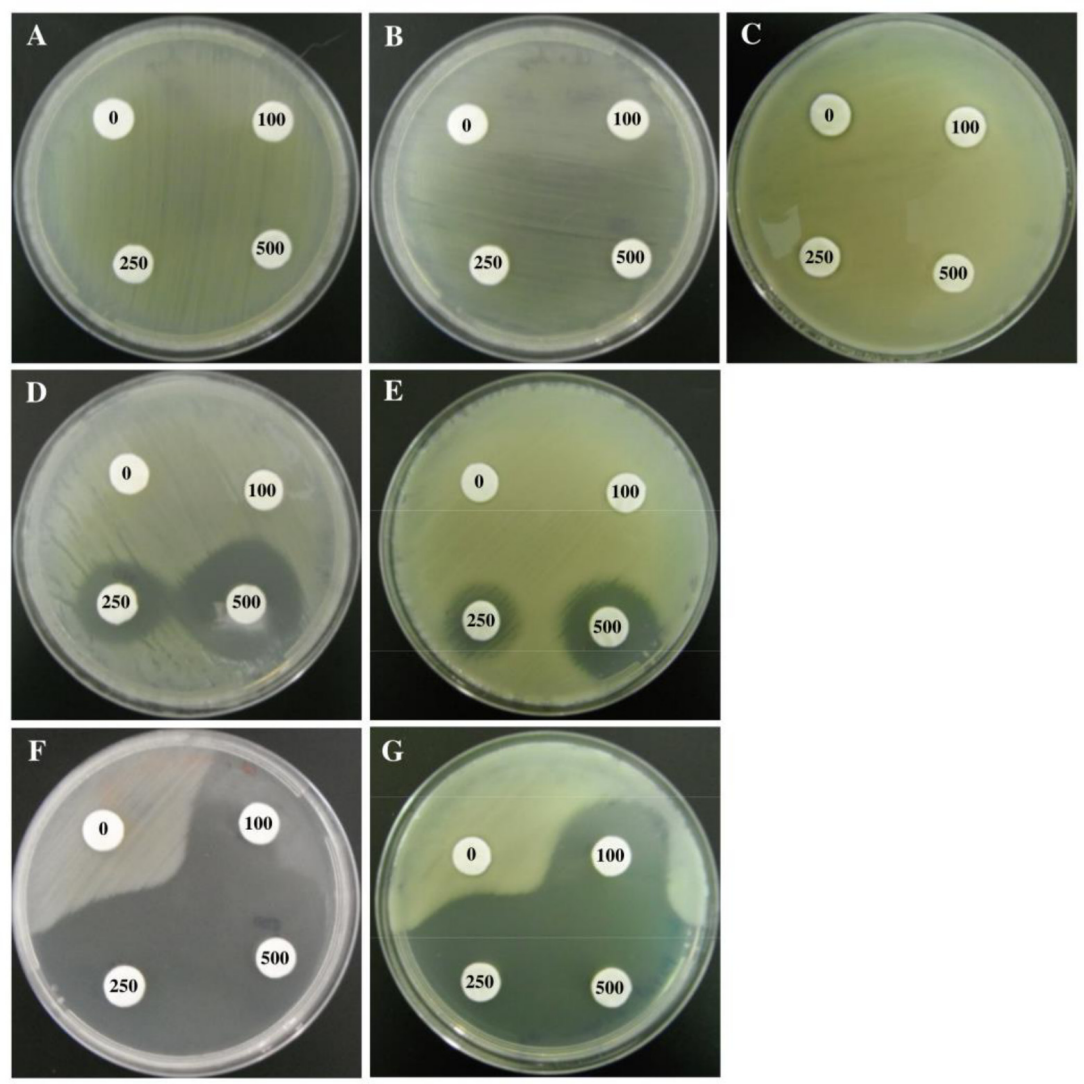
Growth of strains *O*. *tritici* SCII24 (A), *Acr3_1* mutant (B), *Acr3_2* mutant (C), *arsB* mutant (D), *arsB/Acr3_2* mutant (E), *arsB/Acr3_1* mutant (F) and *arsB/Acr_1/Acr_2* mutant (G) on LB medium containing filter discs saturated with diverse arsenite concentrations, after 2 days of incubation at 35°C. Numbers inside filters indicate the corresponding metal concentration used in each filter (0 mM, 100 mM, 250 mM and 500 mM As(III)).

To functionally characterize the interplay between the products of the resistance determinants, mutated and non-mutated strains were tested for arsenite resistance. Bacterial growth was evaluated in solid medium LB with As(III) concentrations ranging from 0 mM to 50 mM (Fig 2). *O*. *tritici* SCII24^T^ and *Acr3_1* or *Acr3_*2 mutants were able to grow up to 50 mM of arsenite. However, they showed a partial inhibition at very high concentrations of As(III) (i.e. > 20 mM). On the other hand, *arsB* and *arsB/Acr3_2* mutants exhibited lower resistance capacity than the wild strain, since they were unable to grow at As(III) concentrations above 5 mM. Moreover, at low As(III) concentrations (< 5 mM) these mutants showed lower survival rates than the wild-type strain. The double *arsB/Acr3_1* or triple *arsB/Acr3_1/Acr3_2* mutants were not able to grow above 1 mM As(III), being the most arsenite sensitive mutants obtained. Differences in As(III) sensitivity between wild-type *O*. *tritici* SCII24^T^ and mutants were also evaluated in Petri dishes, by growing strains in solid LB medium and by using a filter disk assay (Fig 3). The comparison of the inhibition halos revealed that *arsB/Acr3_1* or *arsB/Acr3_1/Acr3_2* mutants were the most As(III) susceptible strains.. Single *arsB* and double *arsB/Acr3_2* mutants also showed As(III) sensibility, particularly in the presence of discs saturated with concentrations higher than 250 mM, although not as evident as for the *arsB/Acr3_1* or *arsB/Acr3_1/Acr3_2* mutants.

### Arsenite uptake assays

**Fig 4:**
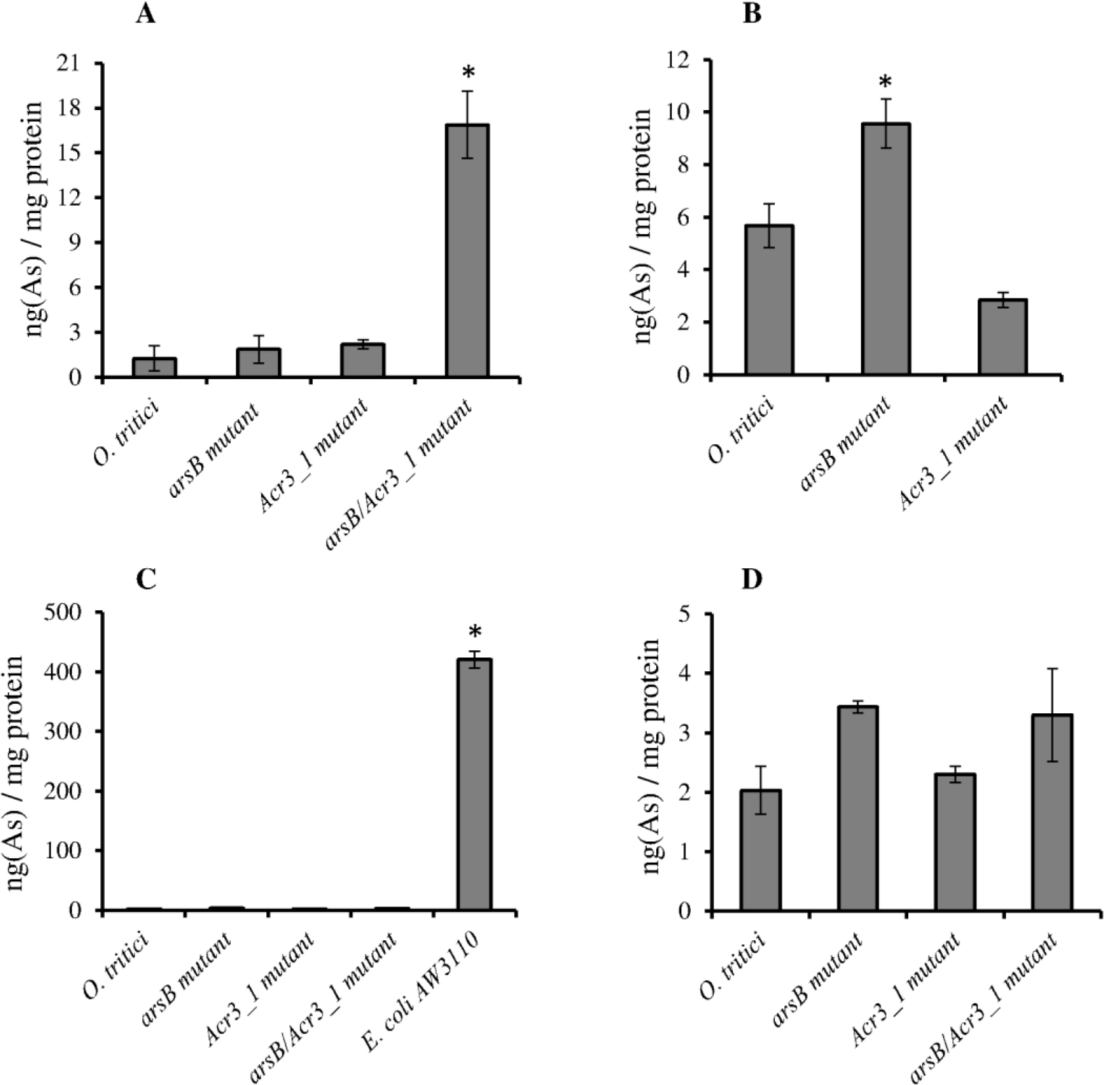
Arsenic uptake by *O*. *tritici* type strain and mutants. Exponential growing cells were exposed to (A) 1 mM,(B) 5 mM arsenite and (C; D) 5 mM arsenate for 3 h. Data shown are the mean values (± standard deviations) obtained from three independent experiments. (*) symbol above bars indicate significant differences between samples (*P* < 0.05).

**Fig 5:**
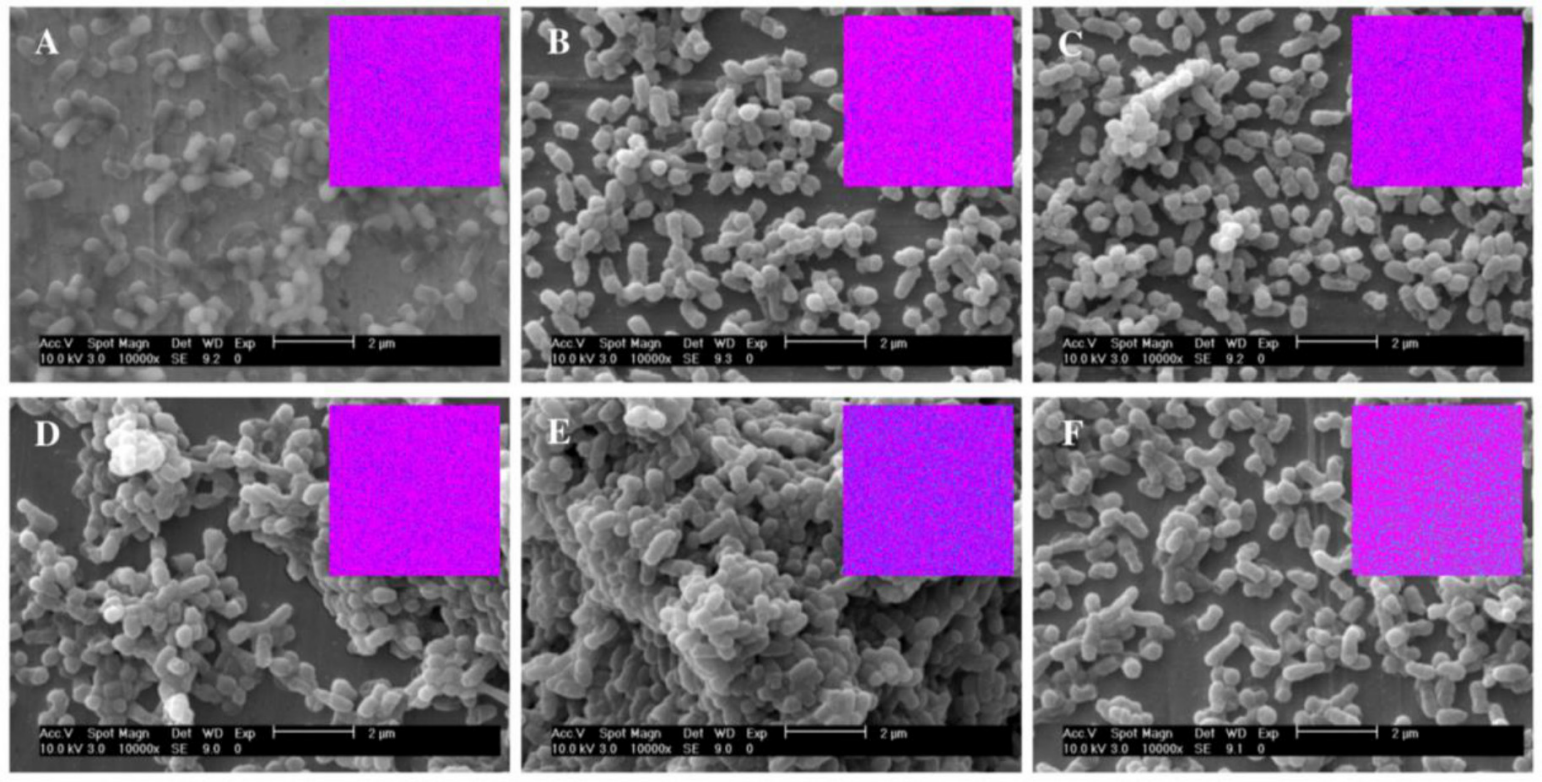
SEM micrographs of arsenic uptake by *O*. *tritici* type strain and double *arsB/Acr3_1* mutant. Exponentially grown *O*. *tritici* incubated for 3 h in the presence of 1 mM As(III) (B) and in the presence of 5 mM As(V) (C), and exponentially grown *arsB/Acr3_1* mutant incubated for 3 h in the presence of 1 mM As(III) (E) and in the presence of 5 mM As(V) (F). Figures A and D are controls where *O*. *tritici* and the double mutant were grown without metal, respectively. Small charts inscribed inside represent a 50x50 μm^2^ area of the elemental map distribution of arsenic inside the cells (where blue corresponds to the presence of As).

The capacity of mutated and non-mutated strains to accumulate arsenic was evaluated by exposing them to 1 mM or 5 mM of As(III) for 3 hours (Fig 4). These assays were only performed with the wild-type *O*. *tritici* SCII24^T^, the single *arsB* and *Acr3_1* mutants and the double *arsB/Acr3_1* mutant, because the results obtained from the arsenite resistance assays indicated no function for *Acr3_2* gene. In the presence of low arsenite concentrations (1 mM) the double *arsB/Acr3_1* mutant was able to accumulate up to 17 ng(As)/ng protein, while the wild-type *O*. *tritici* and the single *arsB* or *Acr3_1* mutants accumulated only about 2 ng(As)/mg protein. After the accumulation tests, all cells showed viability. However, in assays with higher concentrations of arsenite (5 mM), *arsB* mutant accumulated up to 10 ng(As)/mg protein. Double *arsB/Acr3_1* mutant was not tested in this condition since this mutant could not resist concentrations above 1 mM As(III). Both wild-type *O*. *tritici* and the double *arsB/Acr3_1* mutant did not change their cell morphology at low arsenite concentrations (Fig 5). Moreover, the higher capacity of this double mutant to accumulate arsenic was visible in the elemental map distributions (small inserts in Fig 5) where the density of the As-specific signal was higher.

### Arsenate uptake assays

Wild-type *O*. *tritici*, the single *arsB* or *Acr3_1* mutants and the double *arsB/Acr3_1* mutant were also tested for their capacity to accumulate arsenic by subjecting them to 5 mM of As(V) for 3 hours (Fig 4). In this case, an arsenic sensitive strain, *E*. *coli* AW3110, was used as control and its capacity to take up arsenate was also evaluated. Results showed a significant difference concerning the values of arsenic accumulated by *E*. *coli* AW3110, *O*. *tritici* and mutants. *E*. *coli* accumulated about 140-fold more arsenic [420 ng(As)/mg protein] than *O*. *tritici* and its mutants that accumulated only about 3 ng(As)/mg protein. It was also possible to observe that no morphological changes were visible for *O*. *tritici* and *arsB/Acr3_1* mutant grown in the presence of As(V) when compared with assays without exposition to the metalloid (Fig 5). In contrast, numerous *E*. *coli* AW3110 cells were lysed or degraded when grown in the presence of As(V) (data not shown). The elemental map distribution of As for the wild-type strain and *arsB/Acr3_1* mutant are very similar in the cells incubated with and without metal, indicating that the concentration of the accumulated As(V) is lower than the detection limit of the EPMA equipment.

## Discussion

*O*. *tritici* SCII24^T^ can be considered one of the most arsenic resistant microorganisms ever reported [11]. In this organism, the two *ars* operons (*ars1* and *ars2*) previously characterized comprise a large number of genes related to the bacterial ability to resist arsenic [11]. Among these genes, *arsB* and *Acr3_1* encode two different arsenite efflux pumps.

The main objective of this work was to design and construct a strain with abilities to resist and accumulate arsenic in order to be used as a tool for bioremediation. Such a strain should resist high arsenic concentrations, should have an increased ability to take up arsenic ions from the environment and have a reduced efflux of arsenite. Several studies have shown that the inactivation of metal efflux proteins resulted in strains with enhanced metal bioaccumulation ability, which could be seen as a useful tool to include in a bioremediation strategy [11,20,21]. In this sense, the present study was designed in order to inactivate in *O*. *tritici* SCII24^T^ all arsenic efflux pumps to obtain a mutant with reduced capacity to extrude arsenite and therefore, able to keep this metal sequestered into cells.

In the first step of this work, the draft genome sequence of *O*. *tritici* SCII24^T^ was performed and an additional probable operon (*ars3*) was identified, with a genetic arrangement highly similar to *ars2*. The genes considered most likely responsible to encode for proteins able to pump arsenite, conferring resistance to arsenic ions, were the *arsB*, *Acr3_1*, and *Acr3_2* genes.

Six different mutants (single, double or triple mutants) of *O*. *tritici* were successfully constructed, and their behaviour in presence of arsenic species was evaluated to identify the most efficient arsenic bioaccumulator. Both, single *Acr3* mutants as well as the wild *O*. *tritici* strain were able to grow at very high As(III) concentrations compared to the other mutants. On the other hand, *arsB* and *arsB/Acr3_2* mutants showed a moderate response to arsenite while *arsB/Acr3_1* and *arsB/Acr3_1/Acr3_2* mutants showed the lowest arsenite resistance ability. Mutation of *arsB* gene had a more expressive effect on the bacteria than mutation of *Acr3_1* or *Acr3_2* genes, supporting the previous conclusion that ArsB pump is the major contributor to bacterial arsenite resistance in *O*. *tritici* SCII24^T^ [11]. In fact, as *arsB* belongs to a gene cluster *arsRDAcbsB*, ArsB protein pump becomes associated with the ArsA-ATPase, forming a more efficient As(III) efflux pump than ArsB or ACR3 without an associated ATPase [22]. Therefore, the different resistance levels conferred by the presence of *arsB* or *Acr3* alone, comparatively to *arsB* associated with *arsA*, have been explained by the fact that thermodynamically, secondary carriers using membrane potential to export arsenite are less efficient systems than ATPase-associated systems [23].

Additionally, in this *O*. *tritici* SCII24^T^ strain, mutation of the genes coding for the two secondary carrier proteins (ACR3) led to two different arsenite phenotypes. Double mutation of *arsB* and *Acr3_1* genes resulted in a mutant with remarkably low As(III) resistance, while the double mutant of *arsB* and *Acr3_2* genes did not show a more sensitive phenotype compared to the single *arsB* mutant. Moreover, the construction of the triple mutant did not result in a mutant more sensitive to arsenite than the double *arsB/Acr3_1* mutant. Therefore, it seems that the newly found *Acr3_2* gene has no effective role in the mechanism of arsenite resistance in *O*. *tritici*. The results on the As(III) sensitivity of the mutants also support the fact that both genes (*arsB* and *Acr3_1*) play a crucial role in this resistance mechanism. In fact, depending on the surrounding conditions, the genes might play different roles. For As(III) concentrations equal to or higher than 5 mM, *arsB* gene seems to play a crucial role in the resistance ability of the strains and for As(III) concentrations lower than 5 mM, *Acr3_1* gene expression is enough for the resistance of the strains. The presence of multiple copies of the arsenic related genes in individual bacterial genomes is not new, as for instance in *Herminiimonas arsernicoxydans* [24]; *Corynebacterium glutamicum* [2] and *Leptospirillum ferriphilum* [25]. In the most recent years, the rapid development of high-throughput sequencing technology led to the identification of a large number of microbial genomes. Consequently, the access to nearly whole information about the arsenic-related genes of these strains was possible [26]. This bioinformatic analysis often results in data related to abundance and organization of arsenic-related operons but do not provide any evidence about their functionality.

The capacity of strains to accumulate arsenic was different among the mutants. For low As(III) concentrations (1 mM), the double *arsB/Acr3_1*mutant was able to accumulate several times more arsenic than the remaining strains. For higher As(III) concentrations (5 mM), where this double mutant was not able to grow, the single *arsB* mutant was able to accumulate the highest amount of arsenic. Moreover, our double *arsB/Acr3_1* mutant, at a concentration of 7.5 μg/mL protein, was sufficient to remove around 120 ppb of As(III) in 3 hours, being more efficient in arsenic accumulating than other studied strains such as *C*. *glutamicum* [21] or *E*. *coli* [27,28]. Therefore, this bioengineered strain showed a great potential to be used in arsenic bioremediation.

As arsenite and arsenate have different pathways to enter the cell [3], strains were also evaluated regarding their capacity to take up arsenic in the form of arsenate. Cells of *E*. *coli* AW3110 were significantly more efficient at taking up arsenate than wild *O*. *tritici* or mutants (about 140-fold more arsenic). As *E*. *coli* AW3110 is a strain silenced for the *ars* genes, it is unable to extrude arsenic resulting in an hyper-accumulation of arsenate inside cells and, therefore, in hyper-sensitivity to arsenic [7]. However, in the case of *O*. *tritici* SCII24^T^ and their mutants, no notable difference in the capacity of taking up arsenate was observed, and even the *arsB/Acr3_1*mutant, when incubated with arsenate, was not able to accumulate arsenic, despite the inactivation of both efflux proteins. It is possible that in *O*. *tritici* SCII24^T^, and, in consequence, in all mutants, arsenate does not enter the cell via the common phosphate transport system, PitA [3]. Exploring the draft genome of *O*. *tritici*, no Pit system was found. However, the other phosphate-specific transport system, Pst, was identified. It is reported that Pst system is a high-affinity phosphate transport system that differentiates between phosphate and arsenate approximately 100 fold more accurately than PitA [29]. In this strain, most probably, the Pst system discriminates between phosphate and the larger arsenate ions, preventing the entry of As(V), as was already reported for *Halomonas* GFAJ-1 [30]. Nevertheless, other possible mechanisms in *O*. *tritici* capable of detoxifying arsenate cannot be overruled.

Processes of bioremediation have gained increasing interest and biological approaches have been developed to deal with the arsenic contamination problem using bioaccumulation or biosequestration abilities of bacteria [15]. A few genetically modified microorganisms have been developed to yield high accumulation of arsenic by expression of intracellular arsenic-binding proteins [27,31]. Accumulator microorganisms have been used in consortiums with bacteria able to oxidize As(III) to As(V) to bioremediate arsenic more efficiently [21]. In this sense, *O*. *tritici arsB/Acr3_1* mutant is a very interesting alternative tool for arsenite bioremediation, since it gathers several features to deal with arsenic. First, the presence of the arsenite oxidase system AioAB [12] allows a rapid oxidation of As(III) to As(V), which is far less mobile and therefore less toxic than As(III) [32,33]. Secondly, the double mutant would be able to uptake the remaining As(III) from medium which would be maintained inside cells.

In conclusion, this work, with the construction of the different mutants, demonstrated a relationship between the genetic determinants coding for the arsenite pumps and the arsenic resistance. Moreover, it contributed to produce an alternative solution for arsenic removal across a wide range of conditions. Such bacteria could represent an effective way to remove arsenic from contaminated sites. Its in-field application is normally target of some important issues that are being studied to be solved.
